# A long-term follow-up study on otoacoustic emissions testing in paediatric patients with severe malaria in Gabon

**DOI:** 10.1186/s12936-019-2840-9

**Published:** 2019-06-24

**Authors:** Elisa Reiterer, Simon Reider, Peter Lackner, Natalie Fischer, Daniel Dejaco, Herbert Riechelmann, Patrick Zorowka, Peter G. Kremsner, Ayola Akim Adegnika, Erich Schmutzhard, Joachim Schmutzhard

**Affiliations:** 1 Department of Otorhinolaryngology, Medical University Innsbruck, Anichstrasse 35, 6020 Innsbruck, Austria; 2 Department of Internal Medicine, Medical University Innsbruck, Innsbruck, Austria; 3 Department of Neurology, NICU, Medical University Innsbruck, Innsbruck, Austria; 4 Department of Hearing, Speech and Voice Disorders, Medical University, Innsbruck, Austria; 5 Centre de Recherches Médicales de Lambaréné, Albert Schweitzer Hospital (MRUG), Lambaréné, Gabon; 6 Institut für Tropenmedizin, Eberhard Karls Universität Tübingen, Tübingen, Germany

**Keywords:** Severe malaria, Otoacoustic emissions, Hearing loss, Children

## Abstract

**Background:**

In a previous study, severe and cerebral malaria have been connected with acute cochlear malfunction in children, demonstrated by a decrease of transitory evoked otoacoustic emissions (TEOAEs) reproducibility. This study aims to determine whether cochlear malfunction persists for 4 years after recovery from severe malaria in a subset of the previous study’s collective. Follow-up TEOAEs were performed on site (CERMEL, Hôpital Albert Schweitzer, Lambaréné, Gabon) or at the participants’ homes; 33 out of 90 participants included in the initial investigation by Schmutzhard et al. could be retrieved and were re-examined, 31/33 could be included. Of the 57 missing participants, 51 could not be contacted, 1 had moved away, 4 refused to cooperate, and 1 had died.

**Methods:**

As in the initial investigation, participants of this prospective follow-up study were subjected to TEOAE examination on both ears separately. A wave correlation rate of > 60% on both ears was considered a “pass”; if one ear failed to pass, the examination was considered a “fail”. The results were compared to the primary control group. Additionally, a questionnaire has been applied focusing on subsequent malaria infections between the primary inclusion and follow-up and subjective impairment of hearing and/or understanding.

**Results:**

The cohort’s mean age was 9 years, 14 children were female, 18 male. 31 had been originally admitted with severe, one with cerebral malaria. 83.8% of participants (n = 26) presented with a TEOAE correlation rate of > 60% on both ears (the cut-off for good cochlear function); in the control group, 92.2% (n = 83) had passed TEOAE examination on both ears. Recurrent severe malaria was associated with a worse TEOAE correlation rate. Age at infection and gender had no influence on the outcome.

**Conclusions:**

Cochlear malfunction seems to be persistent after 4 years in more than 16% of children hospitalized for malaria. In a healthy control group, this proportion was 7.8%. Yet, the severity of the initial TEOAE-decrease did not predict a worse outcome.

**Electronic supplementary material:**

The online version of this article (10.1186/s12936-019-2840-9) contains supplementary material, which is available to authorized users.

## Background

Malaria, one of the most common tropical infections, can often be fatal, in particular so in children. But if survived, severe and cerebral malaria, have been known to compromise the neurological, language, and cognitive development of the children affected. In 1990, Brewster et al. [1] described cerebral malaria as an important cause of paediatric neurological handicap in the tropics. Fifteen years later, Idro et al. [2] reviewed the existing data on clinical and epidemiological features of cerebral malaria, and found that between 1 and 9% of children suffered from hearing and/or speech impairment after suffering from severe *Plasmodium falciparum* malaria. This association of *P. falciparum* malaria and neurological impairment was also confirmed later [3], with Carter et al. [4] postulating malaria as possibly one of the most common causes of paediatric neurocognitive impairment in tropical countries, maybe even worldwide.

Apart from the already known neurological sequelae, an explicit connection between malaria and acute hearing loss has been established since the beginning of the 1990s. For a long time, quinine had been known to reduce high tone acuity. The onset of the high tone hearing loss was rapid, often went unnoticed, and resolved completely after the end of treatment [1]. Mefloquine has also been reported to cause acute high tone hearing loss, sometimes in combination with tinnitus. The prognosis was worse than in hearing loss secondary to quinine [5–7]. The current gold standard anti-malarial therapy, artemisinin-based combination therapy, has also been assessed with respect to possible ototoxicity. No ototoxic effects could be seen in direct comparison of artemether–lumefantrine with quinine [8]. However a lack of high-quality evidence remains due to the heterogeneity of the studies and data for young children is still missing [9].

Schmutzhard et al. [10] studied hearing loss and malaria for the first time in a murine malaria model. In this animal experiment, C57BL/6j mice were infected with *Plasmodium berghei*. The auditory-evoked brainstem response (ABR) measurement at the peak of the disease showed a significant hearing loss in malaria mice, especially in those with cerebral malaria. The histopathologic analysis of the murine inner ear revealed two intracochlear pathomechanisms as possible major causes for hearing impairment: firstly, apoptosis induced in fibrocytes type 1 in the spiral ligament, and secondly, a disrupted blood labyrinth barrier, suggesting a disturbance of the endocochlear potential [11–13].

Otoacoustic emissions (OAEs) are generated by the outer hair cells within the cochlea. Therefore, they are suitable to assess cochlear malfunction. They have been identified by Kemp in 1978 as sounds in the external auditory canal evoked by inner ear processes [14]. Transitory-evoked OAEs (TEOAEs) are evoked via broad frequency range stimuli (clicks). These clicks consist of a wide bandwidth and stimulate the majority of the basilar membrane, thus producing a contraction of the outer hair cells, which is measured as a frequency-specific sound response. This oscillation is longer than the click itself, which results in a prolonged response signal [15–17]. TEOAE screening is less sensitive than classic pure tone audiometry (PTA) assessment, but can still identify hearing loss in children. In contrast to PTA, TEOAE screening does not require the patient’s active collaboration and is, therefore, the method of choice when examining very young children [16, 18]. OAEs can be measured noninvasively, objectively, and quickly. The device is of relatively small size and thus transportable, and can easily be operated by not audiologically-trained personnel, which is of great importance when working in a rural African setting [19]. Otoacoustic emissions are also stable over a long period of time and, therefore, useful for longitudinal analysis and monitoring of cochlear function [15].

In school-aged children, PTA is the gold standard for hearing assessment. Still, in a rural environment with very limited supplies available, this may be quite challenging as it requires the children’s participation, trained investigators and appropriate facilities. Although the sensitivity and specificity for predicting sensorineural hearing loss in school-aged children is lower than in newborns, TEOAEs are still a simple, efficient, and reliable approach for testing hearing ability in school-aged children [18–21]. TEOAEs were chosen over distortion-product otoacoustic emissions (DPOAEs) because it allows a more reliable and simpler evaluation of hearing loss in young children, particularly in noisy environments [22]. Additionally, TEOAEs are more sensitive to cochlear status than DPOAEs and are even capable to reveal mild to moderate hearing loss [17].

In the past, different studies have applied different pass criteria when assessing otoacoustic emissions [20, 23]. The wave reproducibility has been reported to vary from 50 to 70%. Clinical experience indicates a TEOAE wave reproducibility rate of 60% to be a reliable cut-off parameter for a positive OAE measurement [24–27]. Thus, a wave reproducibility rate < 60% was classified as a failed test result and assumed to correlate with a substantial malfunction of the cochlea, representing a hearing impairment of 20–30 dB or more [15, 17, 24].

In a prospective clinical multicentre trial, malaria infection itself was connected with acute cochlear malfunction in children suffering from severe malaria in Gabon, Ghana and Kenya. The inner-ear function was tested with transient-evoked otoacoustic emissions (TEOAE). More than 40% of children suffering from severe *P. falciparum* malaria failed the TEOAE testing prior to beginning anti-malarial treatment [28]. However, one of the major limitations of this clinical trial was the short follow-up time period of only 28 days. This current study aims to evaluate a long-term follow-up on the otoacoustic passing rates of the Lambaréné/Gabon subgroup included in a 2011 study by Schmutzhard et al. collective 4 years after acute infection. In addition, subjective hearing and understanding was evaluated by means of a questionnaire. The children, if present, older siblings or a guardian were questioned focusing on speech development, response to calls, attentiveness in class and scholastic performance.

## Methods

The objective of the present investigation was to determine whether the above-described decrease of otoacoustic emissions [28] is still present in the examined children 4 years later. The Gabon cohort was chosen as follow-up cohort as it was the biggest in size. The study was approved by the local Ethics Committee.

The follow-up study was designed as a prospective study and aimed to repeat transient OAE measurements in children, which had been included in the prior study in 2011 at the Centre de Recherches Médicales Lambaréné. The achieved results were compared to the primary control population [28, 29]. The patients were evaluated partly at the research facility, partly in their home. The patients and their parent or guardian were also questioned regarding any further malaria infections in between the two assessments (2–4 weeks past infection and follow-up 4 years later) and whether this lead to any further hospitalizations, as well as subjective impairment of hearing and understanding.

The transient-evoked OAE measurements were carried out as described in a previous study by Schmutzhard et al. [28]. TEOAEs measure the functionality of outer hair cells. The outcome of the measurement is limited by surrounding noise and proper sealing of the outer ear canal using the correct ear plug [20]. Five measurements were performed to ensure a correct outcome despite the varying surroundings. Still, one has to keep in mind that in absence of facilities such as a sound-proof examination room, surrounding noise is possible to have an impact on the reliability of the measurement, even with precautionary measures put in place. The cut-off for a passed test was a wave reproducibility rate of 60% or above on at least one ear [24], the reference being the best value out of five. Each measurement consisted of 2080 repetitions, the stimulus being a broadband click ranging from 1 to 4 kHz for 2 ms [28].

As TEOAEs measure are a concrete biological response, false alarms (false positives, type I error) must be taken into consideration [19, 20]. However, false negative measurements are very unlikely, as a properly sealed in-ear microphone should not be able to register a response sound when there is none. Thus, the best out of five measurements was chosen, as it is the physiologically most reliable one. Similarly, one “pass” out of five measurements was sufficient for a “pass” classification.

TEOAE measurements were performed with a Madsen Capella Otoacoustic emissions machine (Otometric, Taastup, Denmark). When measuring TEOAEs, the patient is placed in a relatively quiet environment to ensure a successful evaluation. Then, an ear tip with an integrated miniature loudspeaker microphone is inserted into the patient’s ear. The tip has to be selected carefully, as the right size is important for complete sealing of the ostium of the ear canal [17, 20]. A reduced or absent TEOAE indicates a high probability that the hearing threshold in that frequency range is worse than 20–30 dB HL [15, 17, 24].

TEOAEs are the method of choice when assessing the inner-ear function of young or newly born children and have been implemented in many national hearing screening programs worldwide [23, 30–32]. Studies have shown that TEOAEs, if carried out appropriately, can be a valid option for hearing screening in school-aged children [18–21]. In the primary study the evaluation of the inner ear was performed in children suffering from severe malaria. TEOAEs were chosen because they do not require an active participation by the patient, which could not be expected by the sick children.

For both screening and statistical purposes, TEOAE results are designed as “pass” (> 60% wave reproducibility) or “fail” (< 60% wave reproducibility). Different, and very variable, pass criteria have been proposed for TEOAE screening [23, 27], with varying reference standards across studies [33]. It is suggested that an overall-reproducibility above 60% and a signal-to-noise ratio above 6 dB (a criterion more important when using DPOAEs as a screening device) are reliable cut-off parameters when assessing inner-ear function via otoacoustic emissions [24–27].

A child’s TEOAE examination was considered an overall “fail” if one ear failed to pass, i.e. all five measurements on one ear failed to reach a wave reproducibility of 60% or above. If both ears passed TEOAE examination, i.e. out of five measurements on both ears at least one showed a wave reproducibility of 60% or above, the child was considered a “pass”. The measurements were inserted into a Microsoft Excel 2003 spreadsheet and therein prepared for statistical analysis. Statistical calculation was done using SPSS Statistics for Windows Version 24 (IBM Corp., NY, USA).

The following hypotheses were tested:

i. The proportion of children passing hearing assessment (TEOAE measurement) is higher 5 years after an episode of severe or cerebral malaria than at 4 weeks after that episode.
ii. Residual hearing impairment compared to a healthy control group is detectable in children using TEOAE 5 years after severe or cerebral malaria.

Correlation between wave reproducibility rates of the five consecutive TEOAE measurements within every single patient at the 5-year follow up timepoint was assessed using Pearson’s correlation test.

For the primary analysis (hypothesis 1), proportions of subjects passing the TEOAE test were compared between the disease cohort at the 5-year follow up timepoint and at the four-weeks follow up timepoint using McNemar’s test accounting for the paired characteristic of the data. Additionally (hypothesis 2), Fisher’s exact test was performed to compare the 5-year follow up disease cohort with the control group.

Secondary analysis included the longitudinal assessment of TEOAE results between timepoints 4 and 5 (i.e. on day 14–28, 5 years) using generalized estimated equations (GEE) analysis with a binary logistic model and timepoint as within-subject variable and the outcome of passing TEOAE assessment on at least one side as dependent variable. Contrasts were calculated based on timepoints.

Additionally, a questionnaire consisting of the following three questions was introduced:

At school, do you have difficulty understanding the teacher? When your mother asks you something, do you sometimes not understand what she is saying? Do you have the impression that you cannot hear very well, or that one ear is better than the other? If the child could not answer these three questions, they were modified and asked to the parent or guardian: Has the teacher complained that he/she does not seem to listen at school? Are you under the impression that sometimes your child does not hear you, or do you frequently need to repeat direct questions? Did the child start to talk later than its siblings did? The questions had to be answered with a simple YES or NO.

Information could be gained for 28 of the 31 included participants, and was then inserted into a pie chart (Additional file 1: Figure S1).

## Results

Out of 90 participants included into the study in 2011, 33 could be traced and tested. Out of these, 31 patients could be included in this follow-up study; the identity of 1 patient could not be verified, and measurements could not be completed in 1 case who was visited at home and presented with cerumen on one side. Out of the 57 non-reachable participants, 22 did not leave any contacts, 5 phone numbers were invalid, 3 phone numbers were used by a different person, in 20 cases the contact person was impossible to reach, 1 patient had moved away, in 4 cases the parents refused to cooperate, 1 participant could not be found and was unknown to the inhabitants of his village, and 1 participant had died of cerebral malaria.

Of the 31 participants 14 were female, 17 were male. The mean age at primary admission was 5 years, and, thus, at follow-up 9 years (Tables 1 and 2). Furthermore, no significant difference in TEOAE results could be found between the children measured at home and in the research facility (Additional file 1: Table S1).

**Table 1:** Demographic characteristics of the followed-up subcohort

	n	%
Male	14	45.2
Female	17	54.8
Recurrent malaria	5	16.1
Severe malaria	30	96.8
Cerebral malaria	1	3.2

**Table 2:** Demographic and clinical characteristics of the follow-up subcohort at presentation compared to the original cohort (p values were calculated using either t tests for continuous variables or Fisher’s exact test for dichotomous variables)

Parameter	Units	Original cohort				Follow up subcohort				p value
		N	%	Mean	SD	N	%	Mean	SD	
Age	Years	90	–	4.2	2.4	31	–	4.6	2.4	0.99
Pulse	/min	94	–	124.1	21.8	31	–	115.2	27.513	0.84
Temperature	°C	94	–	38.3	1.2	31	–	38.2	2.17	1.00
Respiratory rate	/min	94	–	36.1	10.9	31	–	32.7	10.86	0.94
Haemoglobin	g/dL	94	–	8.9	2.3	30	–	18.25	49.09	0.83
Platelets	1000/µL	94	–	101.6	87.3	30	–	95.93	58.87	0.90
White blood cells	1000/µL	94	–	9.3	6.2	30	–	9.53	5.99	1.00
Glucose	mmol/L	78	–	4.8	1.7	21	–	5.5	2.74	0.99
Creatinine	g/L	75	–	35.1	64.5	24	–	22.46	8.541	0.80
Bilirubin	g/L	78	–	132.0	827.5	23	–	33	36.9	0.05
ALT	U/L	78	–	35.7	36.3	24	–	32.3	39.95	0.94
Haemoglobinuria	% included patients	93	2.2	–	–	30	0	–	–	1
Respiratory distress	% included patients	93	6.4	–	–	30	0	–	–	0.33
Deep breathing	% included patients	94	2.1	–	–	31	0	–	–	1
Severe vomiting	% included patients	93	2.2	–	–	30	0	–	–	1
Prostration	% included patients	93	20.4	–	–	30	3.2	–	–	0.1
Coma	% included patients	93	4.3	–	–	30	0	–	–	0.57
Repeated generalized seizures	% included patients	93	7.5	–	–	30	3.3	–	–	0.68
Jaundice	% included patients	93	4.3	–	–	30	0	–	–	0.57
Severe anaemia	% included patients	93	8.6	–	–	30	0	–	–	0.20
Hypoglycaemia	% included patients	93	3.2	–	–	30	3.3	–	–	1
Cerebral malaria	% included patients	93	6.4	–	–	31	3.3	–	–	0.68

The cohort included at follow-up is however only partially representative for the malaria collective as a whole, as only one child who had initially suffered from cerebral malaria could be included, which accounts for 3.2% of the followed-up cohort; initially, 6.4% of all included children had experienced cerebral malaria. In addition, it has to be taken into consideration that it is not known how many children initially hospitalized with cerebral malaria even survived up to follow-up.

At follow-up, 84% (n = 26) of children presented with a TEOAE wave reproducibility of > 60% on both ears. 16% (n = 5) presented with a TEOAE wave reproducibility rate of < 60% on at least one ear. The mean wave reproducibility rate on both ears of all followed-up children was 84% with a standard deviation of 15.

In 2011, on admission to the hospital 58.5% of children (n = 94) presented with a TEOAE wave reproducibility rate of > 60% on both ears. 41.5% of included participants showed a TEOAE wave reproducibility rate of < 60% in the acute phase of disease. At 2–4 weeks after admission, the number of participants with a TEOAE wave reproducibility rate > 60% on both ears had risen to 65% (n = 66). At the same time-point, 35% presented with a TEOAE wave reproducibility rate < 60%.

When comparing the measurements of 14–28 days after admission (endpoint of the initial investigation) and follow-up, the mean TEOAE wave reproducibility rates of 11 of the 31 included participants improved, while those of 6 patients worsened. However, 4 of them decreased only slightly and within the tolerance of the measurement (Table 3). In the control group, 92% (n = 83) showed a TEOAE wave reproducibility rate of > 60% on both ears; 8% (n = 7) presented with a TEOAE wave reproducibility rate < 60% on at least one ear. The mean wave reproducibility rate on both ears was 87.11% with a standard deviation of 12.99.

**Table 3:** Descriptive statistics of TEOAE wave reproducibility rates for all locations (left ear, right ear, and mean rates of both ears)

Timepoint	N	Minimum	Maximum	Mean	Std. deviation
At admission					
Right ear	22	0	96	63.55	31.202
Left ear	13	9	95	61.62	32.201
Mean of both ears	23	0.00	95.00	62.0217	29.29609
Valid N (listwise)	12				
1 day after admission					
Right ear	21	4	98	72.71	24.243
Left ear	23	− 28	97	58.78	41.120
Mean of both ears	27	2.00	97.00	66.4259	29.06018
Valid N (listwise)	17				
7 days after admission					
Right ear	20	13	97	69.60	25.800
Left ear	17	− 10	96	50.29	39.099
Mean of both ears	24	3.00	97.00	63.3542	26.40054
Valid N (listwise)	13				
2–4 weeks after admission					
Right ear	14	25	96	74.29	21.995
Left ear	12	45	97	80.00	15.285
Mean of both ears	17	35.00	96.00	79.6765	15.99799
Valid N (listwise)	9				
Follow-up					
Right ear	31	47	99	84.94	15.665
Left ear	31	27	98	82.90	18.659
Mean of both ears	31	37.50	98.00	83.9194	14.78067
Valid N (listwise)	31				

Listed are the minimum, maximum and mean wave reproducibility rate measured per location and timepoint, at each timepoint of the initial study and follow-up. N stands for the number of individual measurements taken at each timepoint. p-values for these comparisons are included in Fig. 3a

**Fig. 1:**
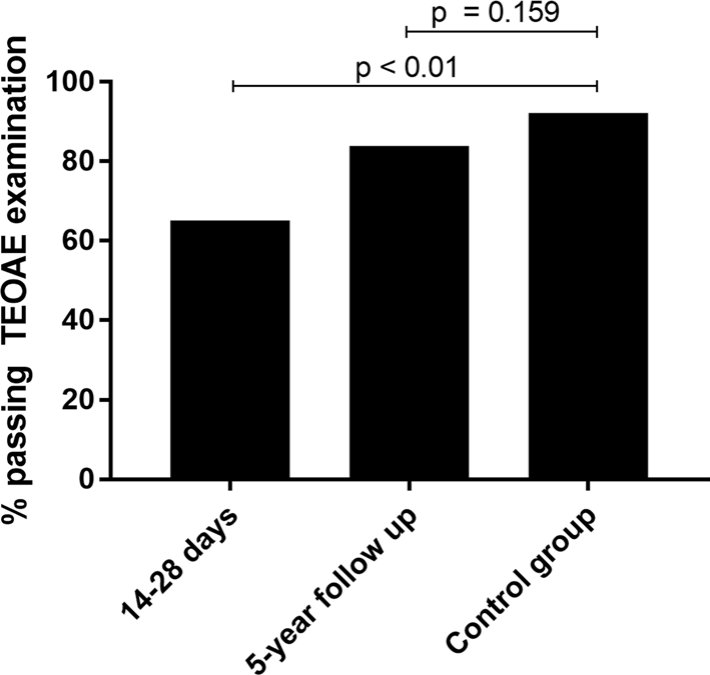
Shows the percentage of participants who presented at follow-up with a TEOAE wave reproducibility rate of < 60% on at least one ear compared to a healthy control group. Statistical significance was assessed using Fisher’s exact test (p value = 0.159)

**Fig. 2:**
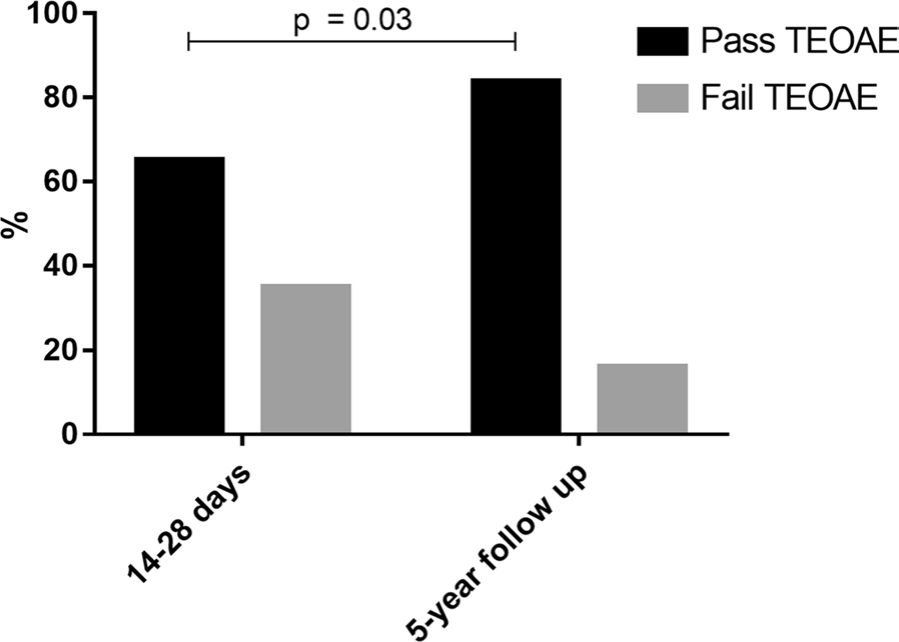
Shows the overall pass rates in black and the overall fail rates in grey at timepoint 4 (14–28 days after admission) compared to the overall pass rates and fail rate at the followed-up measurement (p value from GEE model = 0.189 with sequential Sidak correction)

The study population and the healthy control group did not differ significantly in the rate of passed TEOAE examination (p = 0.159, one-sided Fisher’s exact test; Fig. 1; Table 4). Comparing the 31 followed-up participants to the collective of patients examined at 14–28 days after admission (i.e. after having recovered from the acute malaria infection), 65% (n = 45) had passed TEOAE screening at 2–4 weeks after admission, while at follow-up this number had mounted to 84% (n = 26). In a healthy control group, 90% passed TEOAE examination. Figures 1 and 2 illustrate these situations: Fig. 1 compares the results at both timepoint one and follow-up with the control group, while Fig. 2 highlights the change in TEOAE pass rates between timepoint 4 and follow-up (Table 4). There was a significant difference in passing percentage between the patient cohort at timepoint 4 and the control cohort (p < 0.01, Fisher’s exact test). When comparing the control group to the followed-up cohort, this gap almost closes (p = 0.159, Fisher’s exact test) (Fig. 1). Pass rates at the 5-year follow-up timepoint were significantly higher than at timepoint 4 (Fig. 2, p = 0.03, GEE model).

**Table 4:** Proportion of participants passing TEOAE examination at timepoints 4 and 5 respectively, compared to a healthy control group

	Passed TEOAE examination	Mean wave reproducibility (mean of both ears ± standard deviation)
14–28 days after admission	65.15% (43/66)	79.7 (± 16.00)
5-year follow up	83.87% (26/31)	83.92 (± 14.78)
Control group	92.22% (83/90)	87.11 (± 12.99)

The difference in pass rates between timepoints 4 and 5 was statistically assessed using a GEE model (accounting for repeated measures over time, sequential Sidak corrected p = 0.03 when only comparing the last two timepoints, p = 0.19 when adjusting for multiple comparisons for all 5 timepoints). The difference in mean wave reproducibility was statistically significant between timepoint 4 and the healthy control group (student’s t-test, p < 0.01). The differences between timepoint 4 and 5 and between timepoint 5 and the control group were not significant (p = 0.22 and 0.25, respectively)

**Fig. 3:**
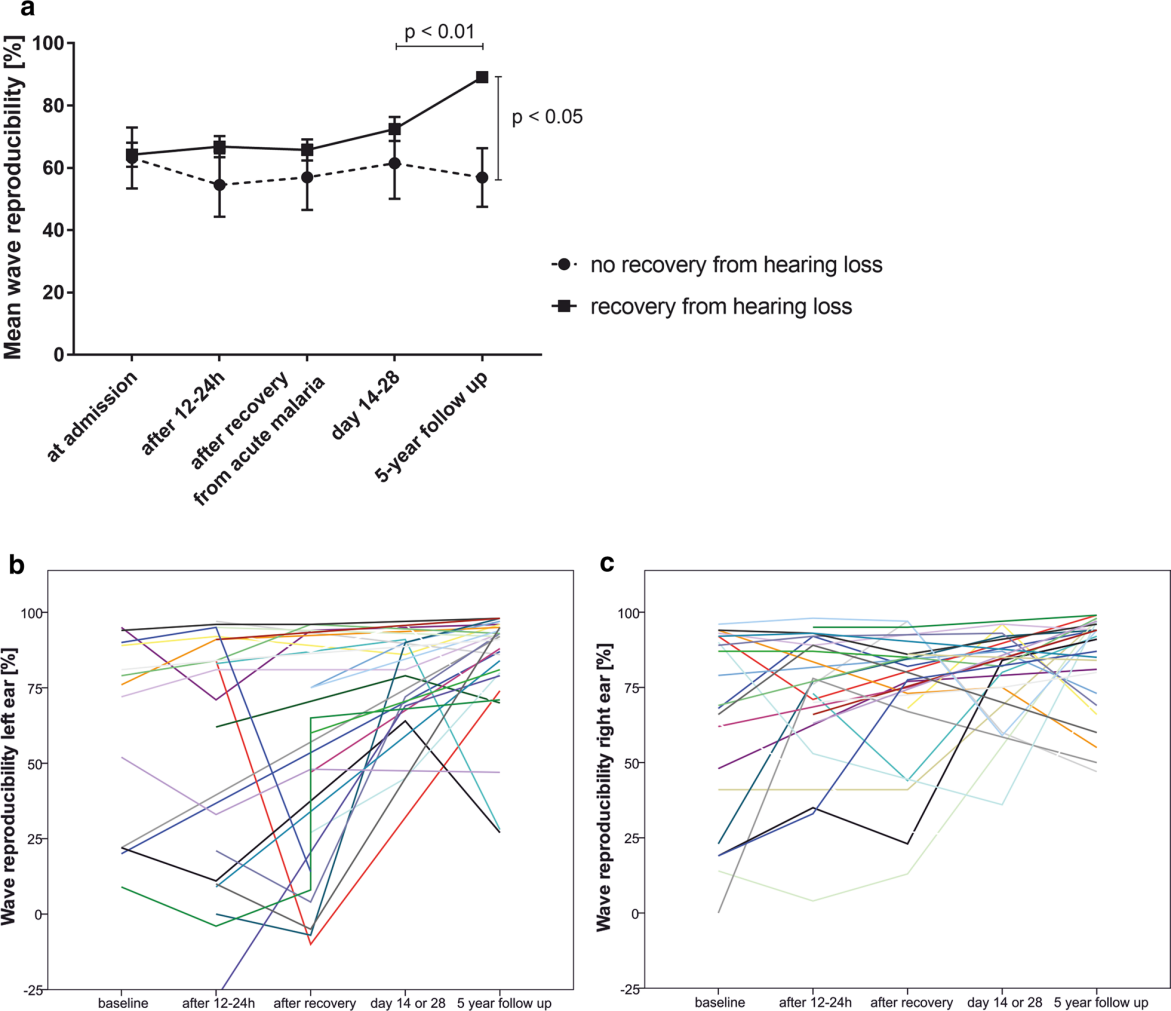
**a** Visualizes a model prediction of the wave reproducibility rates of the patients who had recovered from cochlear damage secondary to acute *P. falciparum* malaria at follow-up versus those who had not. Between day 14 and 28 after admission and follow-up, wave reproducibility rates increased in the recovery group significantly. (p < 0.01, GEE model with sequential Sidak correction). This trajectory illustrates two findings: not all children recover, and recovery occurs between 1 month (timepoint 4) and 5 years (follow-up). **b**, **c** A longitudinal analysis of all measurements (timepoints 1–5) of the followed-up cohort, illustrating that most children recover from cochlear damage, but not all. A distinct dip in wave reproducibility either at 12–24 h after hospitalization or after recovery (7 days later) can be seen

Finally, a longitudinal comparison of all wave reproducibility measurements analysed for each ear separately was done (Fig. 3b, c). A model prediction of the wave reproducibility rates of the subcohort that had recovered at follow-up versus those who did not was then calculated (Fig. 3a). The difference in wave reproducibility rates was not yet significant up to timepoint 4. Between timepoint 4 and 5, wave reproducibility rates increased in the subset of patients who showed recovery at timepoint 5 (p < 0.01, GEE model with sequential Sidak correction).

The participants and/or their guardians were questioned regarding any further malaria infection after the initial testing, as recurrent malaria infection after the initial screening might have negatively influenced the expected outcome. Out of the participants who passed TEOAE examination, 42% (n = 11) never again contracted malaria, while 39% did (n = 10). 3 of them were hospitalized at least once more due to malaria. Out of the 5 participants who did not pass TEOAE testing, 1 had again been hospitalized for malaria; none had suffered from a malaria infection that did not require hospitalization. One participant in each group suffered from recurrent fevers without being tested for malaria. Finally, 5 participants were not able to provide clear information regarding malaria infections.

On the subjective impairment of hearing and understanding questionnaire, clear information could be gained for 28 of the 31 participants; in 1 case the child was not able to provide coherent answers, and in 2 cases no information could be collected (all 3 children were not accompanied). Twelve out of 28 stated to have subjective problems with hearing and understanding. Two had started to talk significantly later than their older siblings. 15 did not describe any of these issues.

Out of the 5 participants with a TEOAE wave reproducibility rate < 60%, 2 did not describe any problems with hearing and understanding. In the group which passed the TEOAE testing, 8 still reported subjective impairment of hearing and understanding.

## Discussion

The majority of included children recuperated most of their cochlear capacity 5 years after experiencing a compromised inner ear function following a severe malaria infection. The followed-up cohort showed no significant difference in outcome compared to a healthy control group. At the previous timepoint (14–28 days after admission), the proportion of patients passing TEOAE examination was significantly lower than in the control group. These findings suggest that, while malaria was associated with cochlear malfunction up to 4 weeks after acute infection, this deficit regressed in a majority of patients during a period of 5 years. However, in 16% a persistent malfunction of the cochlea (shown by a decrease in TEOAEs) is still detectable 5 years after recovery from acute malaria infection. The small sample size (n = 31) has to be considered a major hindrance interpreting the presented data.

Even though the inner ear function improved significantly in 5 years with 83.87% of participants showing positive TEOAEs on both ears (at admission only 58.5% did so), the outcome is still unfavourable compared to the healthy control group (92.22% passing TEOAE examination). Interestingly, the OAE rate of the followed-up group levelled at approximately 84%. Dejaco et al. [29] compared the effect of the recruitment location—in-hospital versus community—on the OAE rates. A significant difference between the two locations could be shown. The in-hospital group showed significantly lower levels at 84%, a passing figure which could be found in the follow-up group as well. This coincidence needs to be examined in further studies. However, no significant difference in the outcome of the children evaluated at home and those evaluated in the research facility could be found in this study. Still, more failed measurements were taken in the research centre. This could be attributed to the elevated level of noise in a public health facility (Additional file 1: Table S1 and Figure S2).

When examining the mean TEOAE wave reproducibility rates on both ears, most of the 31 participants either improved or did not change from 14 to 28 days post admission to follow-up. However, in two cases the mean wave reproducibility rate dropped dramatically, one of whom has been repeatedly hospitalized with malaria. This raises the question whether malaria infections predispose to more severe or even permanent inner-ear damage following second-hit infections [2, 4, 34].

Interestingly, even though their inner-ear function was measured to be intact, a considerable number of children, and often their chaperone too, still described problems with hearing and understanding in everyday life. Transitory-evoked otoacoustic emissions (TEOAEs) have been chosen to test the childrens’ inner-ear function. Otoacoustic emission screening provides an uncomplicated, quick and easy way of diagnosing cochlear hearing loss in children that can be used in almost any setting.

The only possible limitations are scarcity of electric supply or loud background noise. As a major downside, TEOAE examination is not equivalent to pure-tone audiometry regarding sensitivity and in-depth analysis of hearing loss; however, an inner-ear deficit of 20–30 dB can be detected [15, 17, 24]. While pure-tone audiometry remains the standard for acoustic screening in children older than 6 years [29], TEOAE examination should be considered a practical and convenient option when screening children of all ages for inner-ear deficits in remote and rural settings such as sub-Saharan African villages.

Several limitations stem from the nature of a follow-up study. First, the main limitation is the selection bias expected to stem from within the followed-up cohort regarding the outcome of severe versus cerebral malaria. The results of this study underestimate the real impact of severe and cerebral malaria on cochlear function as only one participant with cerebral malaria could be traced and included in the follow-up. Cerebral malaria had initially proved to be the strongest outcome-determining factor [28]; a higher number of included cerebral malaria patients should therefore elevate the percentage of failed tests.

A further limiting factor of this study is the small cohort size of both the participant and the control group: a power analysis showed that a group size of at least 258 individuals per group is necessary to report a result at alpha = 0.05 at power = 80%. The results provide an estimate of the expected effect size (roughly 0.5), but fail to reach significance. The short duration of the initial study period (only 14–28 days) in 2011 might constitute a second limitation [28].

As TEOAEs only assess inner-ear function, many other causes of childhood hearing impairment have to be taken into consideration when implementing questionnaires. Additionally, the language barrier when interviewing children in a language that is often not their mother tongue has to be kept in mind.

Using the same control group as in the initial study can be counted as a limitation, foremost because age-matching proved difficult as the control group had been designed to fit the initial study. Instead, the whole representative collective was reused as a control cohort instead of age-matching every followed-up patient with a child included in the control group [28, 29].

As this study aims to follow up a predefined clinical cohort, it was adamant to follow the exact procedures of the initial study. Only initially included patients could be followed up, therefore limiting the possible overall number of children included.

Another factor is the usage of TEOAEs to screen schoolchildren. TEOAEs are routinely used for screening neonates and very young children. As stated in the introduction, their use in pre-school and school-age children is not very common, as children of that age are usually able to perform a standard PTA screening, but still possible [18, 21]. Pure-tone audiometry requires trained personnel as well as a properly equipped portable facility, both of which are rarely available when working in rural to remote tropical environments. Furthermore, the primary study had been designed to evaluate the inner ear of severely sick children not being capable of performing a demanding PTA. In the setting with an unresponsive patient—like patients with acute severe malaria—TOAEs are very likely to provide a valuable result, whereas PTA is not performable.

Another possible limitation is the possibility of false-positive TEOAE screenings. This technical variability of results (wave reproducibility) is another reason why the best value out of five has been selected [19, 20]—it is a good way to rule out false-positive measurements. It needs to also be pointed out that, even though variability is high, the correlation between replicates of wave reproducibility is strong (left ear: mean Pearson’s r = 0.66, p < 0.01; right ear: mean Pearson’s r = 0.67, p < 0.01).

*Plasmodium falciparum* causes over 200 million malaria cases per year; therefore, persisting hearing impairment in even a small number of individuals would still result in an immense number of disabled people with enormous developmental and socioeconomic impact [28, 35]. Given an infection prevalence of 16% among children between the age of 2–10 years in sub-Saharan Africa [36], and an estimated rough 414 million of children between the age of 0–14 living in the same region [37], the sheer size of this public health problem can easily be imagined. Carter et al. [3] estimated that if the impairment of hearing, speech and cognition persists, at least 250,000 children in sub-Saharan Africa would suffer each year from the repercussions of a severe malaria infection. Even single-sided hearing impairment makes children more prone to develop behavioural problems in school, achieve lower grades and show deficits in language development [38–41]. Therefore, even if hearing impairment caused by cochlear malfunction secondary to a malaria infection is not persistent over years, it still can heavily impact speech development if it affects the child during the vulnerable years of language acquisition. To avoid this, once sensorineural hearing impairment has been discovered, small steps could go a long way: for example, children should be addressed directly, with a clear voice and pronounced speech, in class the child should be seated in the front row, parents should read aloud with their children.

## Conclusions

This study indicates that cochlear malfunction due to acute malaria infection regresses in the better part of affected children during a 5-year follow-up period. Still, it also highlights the persistence of cochlear hearing loss in a considerable subpopulation. Cochlear malfunction with resulting hearing impairment secondary to malaria infection is posing a significant public health problem in developing countries that needs to be addressed properly. A large-scale prospective study including standardized questionnaires regarding subjective performance in hearing and understanding would be of importance to determine the full extent of the situation, and to allow measurements to be put in place.

## Additional file

**Additional file 1: Figure S1.** The results of the questionnaire regarding hearing and understanding in absolute numbers (n = 31). **Figure S2.** The wave reproducibility rates of each ear (left and right) are compared according to the setting in which the measurement was taken: at the research facility (CERMEL, Centre des Rechèrches Médicales Lambaréné, n = 15) or at home (n = 16). Statistical significance is assessed using independent samples t-test. **Table S1.** Of the 31 patients that could be included in the follow-up study, 15 were assessed in the research facility and 16 at home.

## Data Availability

The datasets used and/or analysed during the current study are available from the corresponding author on reasonable request.

## Abbreviations

CERMEL: Centre des Recherches Medicales Lambaréné
Corr_le: TEOAE correlation rate on left ear
Corr_ri: TEOAE correlation rate on right ear
Corr_mean: mean TEOAE correlation rate of both ears
DPOAEs: distortion-product otoacoustic emissions
FU: follow-up
kHz: kilo hertz
OAE: otoacoustic emissions
PTA: pure tone audiometry
Tbl.: table
T.point: timepoint
TEOAEs: transitory-evoked otoacoustic emissions

## References

[1] Brewster DR, Kwiatkowski D, White NJ (1990). Neurological sequelae of cerebral malaria in children. Lancet.

[2] Idro R, Jenkins NE, Newton CRJC (2005). Pathogenesis, clinical features, and neurological outcome of cerebral malaria. Lancet Neurol.

[3] Carter JA, Ross AJ, Neville BGR, Obiero E, Katana K, Mung’ala-Odera V (2005). Developmental impairments following severe falciparum malaria in children. Trop Med Int Health.

[4] Carter JA, Mung’ala-Odera V, Neville BGR, Murira G, Mturi N, Musumba C (2005). Persistent neurocognitive impairments associated with severe falciparum malaria in Kenyan children. J Neurol Neurosurg Psychiatry.

[5] Fusetti M, Eibenstein A, Corridore V, Hueck S, Chiti-Batelli S (1999). Mefloquine and ototoxicity: a report of 3 cases. Clin Ter.

[6] Phillips-Howard PA, ter Kuile FO (1995). CNS adverse events associated with antimalarial agents. Fact or fiction?. Drug Saf.

[7] Wise M, Toovey S (2007). Reversible hearing loss in temporal association with chemoprophylactic mefloquine use. Travel Med Infect Dis.

[8] Gurkov R, Eshetu T, Barreto Miranda I, Behrens-Riha N, Mamo Y, Girma T (2008). Ototoxicity of artemether/lumefantrine in the treatment of falciparum malaria: a randomized trial. Malar J.

[9] Ramos-Martín V, González-Martínez C, Mackenzie I, Schmutzhard J, Lalloo DG, Pace C (2014). Neuroauditory toxicity of artemisinin combination therapies—have safety concerns been addressed?. Am J Trop Med Hyg.

[10] Schmutzhard J, Kositz CH, Lackner P, Dietmann A, Fischer M, Glueckert R (2010). Murine malaria is associated with significant hearing impairment. Malar J.

[11] Schmutzhard J, Kositz CH, Glueckert R, Schmutzhard E, Schrott-Fischer A, Lackner P (2012). Apoptosis of the fibrocytes type 1 in the spiral ligament and blood labyrinth barrier disturbance cause hearing impairment in murine cerebral malaria. Malar J.

[12] Wiese L, Kurtzhals JAL, Penkowa M (2006). Neuronal apoptosis, metallothionein expression and proinflammatory responses during cerebral malaria in mice. Exp Neurol.

[13] Lackner P, Burger C, Pfaller K, Heussler V, Helbok R, Morandell M (2007). Apoptosis in experimental cerebral malaria: spatial profile of cleaved caspase-3 and ultrastructural alterations in different disease stages. Neuropathol Appl Neurobiol.

[14] Kemp DT (1978). Stimulated acoustic emissions from within the human auditory system. J Acoust Soc Am.

[15] Whitehead ML, Stagner BB, Lonsbury-Martin BL, Martin GK (1994). Measurement of otoacoustic emissions for hearing assessment. IEEE Eng Med Biol Mag.

[16] Ho V, Daly KA, Hunter LL, Davey C (2002). Otoacoustic emissions and tympanometry screening among 0–5 year olds. Laryngoscope.

[17] Kemp DT (2002). Otoacoustic emissions, their origin in cochlear function, and use. Br Med Bull.

[18] Sabo MP, Winston R, Macias JD (2000). Comparison of pure tone and transient otoacoustic emissions screening in a grade school population. Am J Otol.

[19] Trzaskowski B, Pilka E, Jedrzejczak WW, Skarzynski H (2015). Criteria for detection of transiently evoked otoacoustic emissions in schoolchildren. Int J Pediatr Otorhinolaryngol.

[20] Richardson MP, Williamson TJ, Lenton SW, Tarlow MJ, Rudd PT (1995). Otoacoustic emissions as a screening test for hearing impairment in children. Arch Dis Child.

[21] Prieve BA, Schooling T, Venediktov R, Franceschini N (2015). An evidence-based systematic review on the diagnostic accuracy of hearing screening instruments for preschool- and school-age children. Am J Audiol.

[22] Tzanakakis MG, Chimona TS, Apazidou E, Giannakopoulou C, Velegrakis GA, Papadakis CE (2016). Transitory evoked otoacoustic emission (TEOAE) and distortion product otoacoustic emission (DPOAE) outcomes from a three-stage newborn hearing screening protocol. Hippokratia.

[23] Akinpelu OV, Peleva E, Funnell WRJ, Daniel SJ (2014). Otoacoustic emissions in newborn hearing screening: a systematic review of the effects of different protocols on test outcomes. Int J Pediatr Otorhinolaryngol.

[24] Hoth S (2009). Objective audiological diagnostics. Sprache Stimme Gehör.

[25] Mühler R, Hoth S (2014). Objektive audiologische Diagnostik im Kindesalter. HNO.

[26] Hoth S, Baljic I (2017). Current audiological diagnostics. GMS Curr Top Otorhinolaryngol Head Neck Surg.

[27] Dirckx JJ, Daemers K, Somers T, Offeciers FE, Govaerts PJ (1996). Numerical assessment of TOAE screening results: currently used criteria and their effect on TOAE prevalence figures. Acta Otolaryngol.

[28] Schmutzhard J, Lackner P, Helbok R, Hurth HV, Aregger FC, Muigg V (2015). Severe malaria in children leads to a significant impairment of transitory otoacoustic emissions—a prospective multicenter cohort study. BMC Med.

[29] Dejaco D, Aregger FC, Hurth HV, Kegele J, Muigg V, Oberhammer L (2017). Evaluation of transient-evoked otoacoustic emissions in a healthy 1 to 10 year pediatric cohort in Sub-Saharan Africa. Int J Pediatr Otorhinolaryngol.

[30] Caluraud S, Marcolla-Bouchetemble A, de Barros A, Moreau-Lenoir F, de Sevin E, Rerolle S (2015). Newborn hearing screening: analysis and outcomes after 100,000 births in Upper-Normandy French region. Int J Pediatr Otorhinolaryngol.

[31] Russ SA, White K, Dougherty D, Forsman I (2010). Newborn hearing screening in the United States: historical perspective and future directions. Pediatrics.

[32] Yousefi J, Ajalloueyan M, Amirsalari S, Hassanali Fard M (2013). The specificity and sensitivity of transient otoacustic emission in neonatal hearing screening compared with diagnostic test of auditory brain stem response in Tehran hospitals. Iran J Pediatr.

[33] Nelson HD, Nygren P, Walker M, Panoscha R (2006). Screening for speech and language delay in preschool children: systematic evidence review for the US Preventive Services Task Force. Pediatrics.

[34] Idro R, Kakooza-Mwesige A, Balyejjussa S, Mirembe G, Mugasha C, Tugumisirize J (2010). Severe neurological sequelae and behaviour problems after cerebral malaria in Ugandan children. BMC Res Notes.

[35] Zhao SZ, Mackenzie IJ (2011). Deafness: malaria as a forgotten cause. Ann Trop Paediatr.

[36] WHO (2015). World malaria report 2015.

[37] United Nations DoEaSA, Population Division (2015). World population prospects: the 2015 revision, volume I: comprehensive tables. ST/ESA/SER.A/379.

[38] Bess FH, Tharpe AM (1984). Unilateral hearing impairment in children. Pediatrics.

[39] Lieu JEC, Tye-Murray N, Karzon RK, Piccirillo JF (2010). Unilateral hearing loss is associated with worse speech-language scores in children. Pediatrics.

[40] Brookhouser PE, Worthington DW, Kelly WJ (1991). Unilateral hearing loss in children. Laryngoscope.

[41] Schmithorst VJ, Plante E, Holland S (2014). Unilateral deafness in children affects development of multi-modal modulation and default mode networks. Front Hum Neurosci.

