# Supplementary material for: A long-term follow-up study on otoacoustic emissions testing in paediatric patients with severe malaria in Gabon
Source: Malar J. 2019 Jun 24;18:212. doi: 10.1186/s12936-019-2840-9 (PMC6591898; doi:10.1186/s12936-019-2840-9)
Supplement: Supplementary file 1 — Additional file 1: Figure S1. The results of the questionnaire regarding hearing and understanding in absolute numbers (n = 31). Figure S2. The wave reproducibility rates of each ear (left and right) are compared according to the setting in which the measurement was taken: at the research facility (CERMEL, Centre des Rechèrches Médicales Lambaréné, n = 15) or at home (n = 16). Statistical significance is assessed using independent samples t-test. Table S1. Of the 31 patients that could be included in the follow-up study, 15 were assessed in the research facility and 16 at home. [file 12936_2019_2840_MOESM1_ESM.docx]

**Additional file 1**

**A long-term follow-up study on otoacoustic emissions testing in pediatric patients with severe malaria in Gabon**

Elisa Reiterer^1^, Simon Reider^2^, Peter Lackner^3^, Natalie Fischer^1^, Daniel Dejaco^1^, Herbert Riechelmann^1^, Patrick Zorowka^4^, Peter Kremsner^5,6^, Ayola Akim Adegnika^5^, Erich Schmutzhard^3^, Joachim Schmutzhard^1*^

**Additional Figures**

Figure S1. The results of the questionnaire regarding hearing and understanding in absolute numbers (n=31)


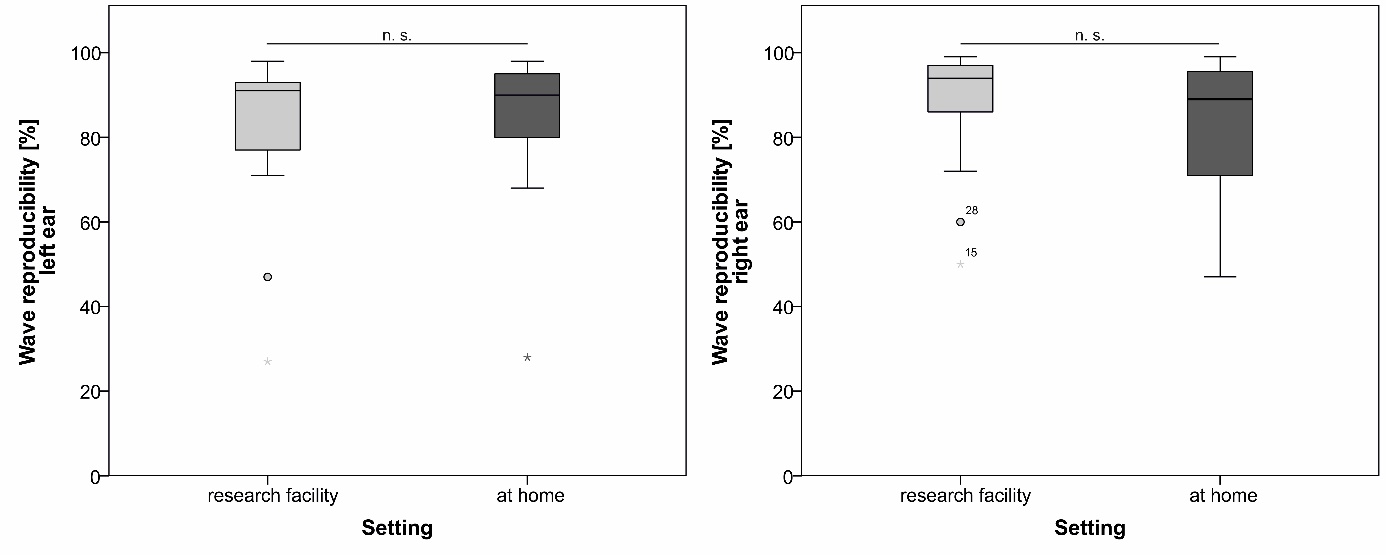
Figure S2. The wave reproducibility rates of each ear (left and right) are compared according to the setting in which the measurement was taken: at the research facility (CERMEL, Centre des Rechèrches Médicales Lambaréné, n=15) or at home (n=16). Statistical significance is assessed using independent samples t-test.

**Additional Table**

Table S1. Of the 31 patients that could be included in the follow-up study, 15 were assessed in the research facility and 16 at home.

|  | **TEOAE pass rates** | | | **best wave reproducibility rate** | | |
| --- | --- | --- | --- | --- | --- | --- |
|  | **facility** | **home** | **p** | **facility** | **home** | **p** |
| **left ear** | 13/15  (86.7 %) | 15/16  (93.8 %) | 0.6 | 81.60  (+/- 20.2) | 84.13  (+/- 17.7) | 0.71 |
| **right ear** | 14/15  (93.3 %) | 14/16  (87.5 %) | 1 | 87.33  (+/- 15.0) | 82.70  (+/- 16.5) | 0.42 |
